# Effectiveness of Active Release Technique With Conventional Therapy in the Management of Lateral Epicondylitis: A Case Report

**DOI:** 10.7759/cureus.50926

**Published:** 2023-12-21

**Authors:** Aditi Nagore, Subrat Samal, Vaishnavi M Thakre

**Affiliations:** 1 Musculoskeletal Physiotherapy, Ravi Nair Physiotherapy College, Datta Meghe Institute of Higher Education and Research, Wardha, IND

**Keywords:** physiotherapy, tennis elbow, active release technique, lateral epicondylitis, ultrasound

## Abstract

The condition known as tendonitis, tennis elbow, lateral epicondylalgia, or lateral epicondylitis affects the radiohumeral joint and causes persistent, severe discomfort in the elbow. It commonly affects those who work in professions that need repetitive forearm motions, like athletes who play on courts, computer operators, and woodworkers. It tends to originate from additional rapid, tiresome, recurring eccentric contractions and activities that grab gliding joints. It commonly affects the dominant hand. This case report describes the author’s seven years of experience living with lateral epicondylitis, including functional disability in day-to-day life, and then physiotherapy management was started. Mill’s and Cozen’s tests were performed, and lateral epicondylitis was confirmed. From 2023, the physiotherapy session, including active release technique and conventional therapy, was taken regularly for four weeks. The outcome measure score of the Visual Analog Scale (VAS) was 7/10 pre-treatment and 2/10 post-treatment, and for the Patient-Rated Tennis Elbow Evaluation (PRTEE) scale, it was 50/100 pre-treatment and 25/100 post-treatment. The results that are reported from this particular case study suggest that physiotherapy has a significant effect in improving muscle strength, increasing range of motion, improving activities of daily living, and enhancing the overall quality of life. The study concludes a physiotherapist is crucial in treating these overuse injuries and returning the patient to their daily activities.

## Introduction

The elbow joint is a hinge joint formed by the articulation of the humerus, radius, and ulna bones. It allows for flexion and extension movements, which are crucial for forearm and hand activities. The joint allows for both pronation and supination movements of the forearm. The common extensor origin over lateral epicondylitis, that is, extensor carpi radialis brevis (ECRB), is the most frequently affected among the extensor digitorum longus (EDL), extensor carpi radialis longus (ECRL), and extensor carpi ulnaris, although they may also be impacted [1]. It is frequently caused by repetitive upper extremity activities like using a laptop, lifting big objects, pronating and supinating the forearm, and vibrating repeatedly [2]. Extensor wrist muscles can become overloaded in the tennis elbow, causing inflammation and degenerative changes in the fibrous tissue, such as tendinosis and microtears. It is estimated that 15.1 instances of lateral epicondylitis occur annually per 10,000 people in the United States. Reportedly, 1%-3% of the general population suffers from this condition [3].

Extremely quick, routine, continuous eccentric contractions and wrist-grabbing activities frequently result in macroscopic and microscopic tears of the ECRB [4]. Usually, the discomfort is tied to a sport or job. The primary signs and symptoms are soreness and a loss of grip strength, which can make everyday activities difficult [5]. It has a clearly defined clinical appearance [6]. The extensor origin muscles' are ruptured by fast and repetitive movement of the wrist and forearm, causing chronic pain and localized inflammation [7]. Therefore, progressive slow, repetitive strengthening exercises increase the load and tension placed on the muscles while improving their strength and tolerance of repeated motion [8].

Tennis elbow can be diagnosed and confirmed through special tests, including Mill's test and Cozen's test, that induce pain, reveal palpable soreness over the lateral epicondyle, and assess resistance during wrist and middle finger extension, as well as passive wrist flexion [9].

The Mill's test involves gently pronating the subject's forearm, fully flexing the wrist, extending the elbow, and then palpating the lateral epicondyle to assess the lateral epicondylitis.

In the second Cozen test, the examiner applies pressure to the lateral epicondyle with the thumb to stabilize the subject's elbow [10]. The patient is then advised to deviate radially while the examiner resists, make a fist, extend their wrist, and pronate their forearm. An excellent sign is abrupt, acute discomfort in the humeral lateral epicondyle. To identify the cause of pain and the third resistance, the epicondyle can be palpated. The EDL and related tendon are stressed during the middle finger extension test when the examiner refuses to allow the third digit to extend over the proximal interphalangeal joint. Pain above the lateral epicondyle of the humerus indicates a positive test.

The repetitive movement caused by overuse or incorrect joint biomechanics can result in microtrauma [11]. This physically bends scar tissue and overloads the healing tissues, activating free nerve endings and producing mechanical nociceptive pain. Proliferating fibroblasts within the tendon will not only produce acute inflammation but also a degenerative or unsuccessful reparative process.

When compared to other scales for pain assessment, the Visual Analog Scale (VAS) for pain evaluation has a high degree of dependability [12,13]. It is also readily available, easy to use, and suitable for any situation. The hand-held Smedley spring dynamometer was used to evaluate grip strength; it is lightweight, portable, easier to use, and produces more accurate results [14].

## Case presentation

A 25-year-old female has been playing badminton since August 2017 and gradually started experiencing pain for two years that is intermittent in nature over the lateral side of the elbow joint. Then slowly her pain got aggravated while doing activities such as writing, typing, holding a pen, and grasping an object. There was swelling present all the time on the forearm and lateral aspect of the elbow joint. After that, she managed the pain by taking painkillers, visited a private clinic at Wardha, and was examined by a physiotherapist. She was diagnosed with lateral epicondylitis. She took regular treatment such as ultrasound, isometric stretches for the wrist joint, and ice-fomentation for two weeks. Then the pain was reduced and home exercises were continued.

In July 2018, the pain again got aggravated, and then she went to an orthopedician at Acharya Vinoba Bhave Rural Hospital in Wardha. The doctor suggested she take some steroid injections, but as she was not willing to take them, the doctor prescribed her one medication, i.e., a combination of diclofenac and metaxalone, and told her to continue with the home exercises and also advised her to wear tennis elbow brace while doing activities. In April 2020, she got an opportunity for a badminton tournament at Pune where her pain got aggravated and was managed with a sturdy, elastic tape that is often composed of a cotton and nylon blend called Kinesio taping and some pain relief spray. After coming back from there, she visited the private clinic at Wardha where treatment was continued for two weeks. Between that, for some personal reason, she was not able to continue her physiotherapy treatment. Now in January 2023, her symptoms got aggravated for which she again visited Acharya Vinoba Bhave Rural Hospital and was further referred for physiotherapy management.

Clinical findings

On examination, swelling was observed in the upper third of the forearm and the extensor origin of the right elbow. On a VAS scale, the patient reported having bearable pain, scoring 7 according to the numerical pain rating scale. A grade 2 tenderness (patient complains of pain and winces) was palpated over the lateral epicondyle of the humerus. A trigger point, which is a substantial knot in the taut bands of the fascia of the skeletal muscles that is extremely irritable, was palpated over the common extensor origin. The patient's written consent was obtained before the therapy procedures began on the first day. Active release technique combined with eight minutes of clinical ultrasound was the specific intervention utilized for the treatment. Over four weeks, the patient had 12 treatments, three times a week, every other day. Compared to the left upper limb, the right upper limb had muscle weakening (Table 1). The range of motion of the right elbow and wrist (the affected side) was severely restricted, and it was painful in every range (Table 2).

**Table 1:** Range of motion pre and post-treatment

Joints	Right (pre)	Right (post)	Left (pre)	Left (post)
Elbow flexion	0-130°	0-140°	0-145°	0-145°
Elbow extension	0°	0°	0°	0°
Pronation	0-75°	0-85°	0-80°	0-80°
Supination	0-70°	0-80°	0-80°	0-80°
Wrist flexion	0-65°	0-75°	0-75°	0-75°
Wrist extension	0-50°	0-60°	0-75°	0-75°
Radial deviation	0-10°	0-20°	0-25°	0-25°
Ulnar deviation	0-25°	0-35°	0-30°	0-30°

**Table 2:** Manual muscle testing pre and post-treatment

Upper limb joints	Right (pre)	Right (post)	Left (pre)	Left (post)
Elbow flexion	4/5	4/5	4/5	4/5
Elbow extension	3/5	4/5	4/5	4/5
Pronation	3/5	4/5	4/5	4/5
Supination	3/5	4/5	4/5	4/5
Wrist flexion	3/5	4/5	4/5	4/5
Wrist extension	3/5	4/5	4/5	4/5
Radial deviation	3/5	4/5	4/5	4/5
Ulnar deviation	3/5	4/5	4/5	4/5

Timeline

Table 3 shows a description of the timeline.

**Table 3:** Timeline AVBRH: Acharya Vinoba Bhave Rural Hospital.

Date of symptoms arousal and visit to a private physiotherapist	August 2017
Visited AVBRH to the orthopedician	July 2018
Again visited physiotherapist	April 2020
Symptoms got aggravated and revisited a physiotherapist at AVBRH	February 2023
Physiotherapy session started	February 2023

Physiotherapy management

**Figure 1:**
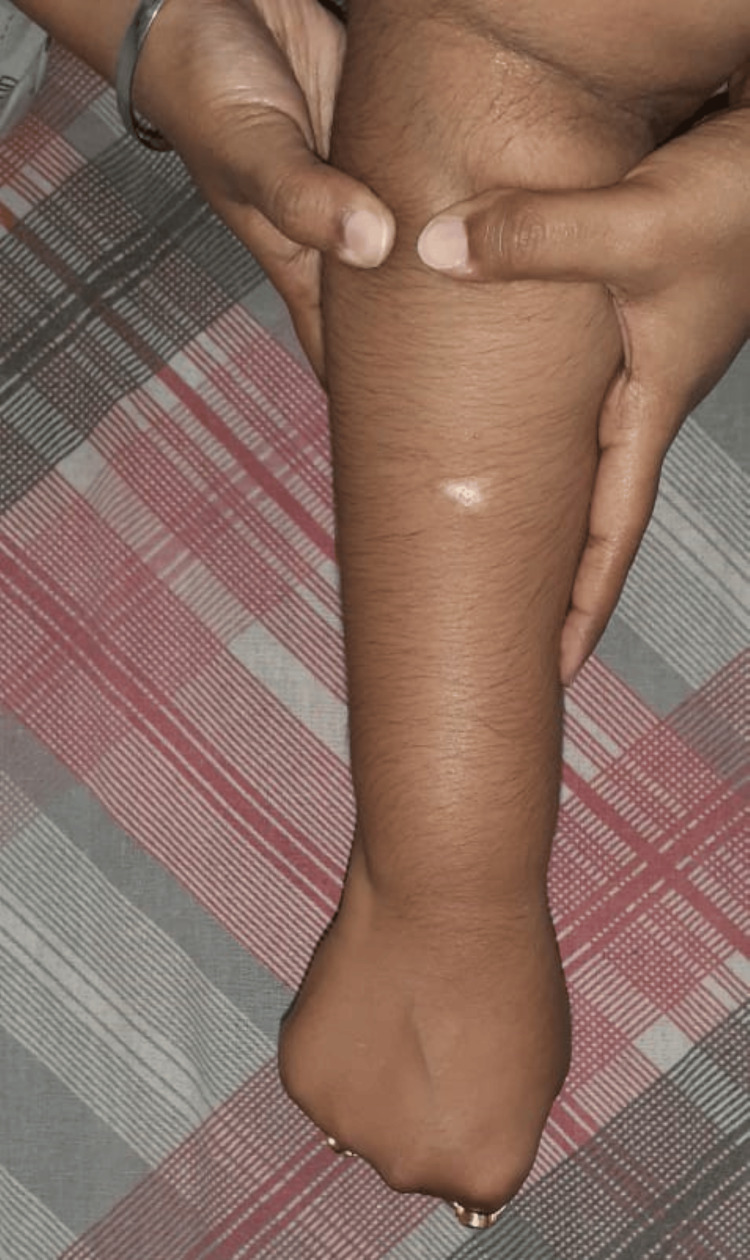
Patient performing active release technique

**Figure 2:**
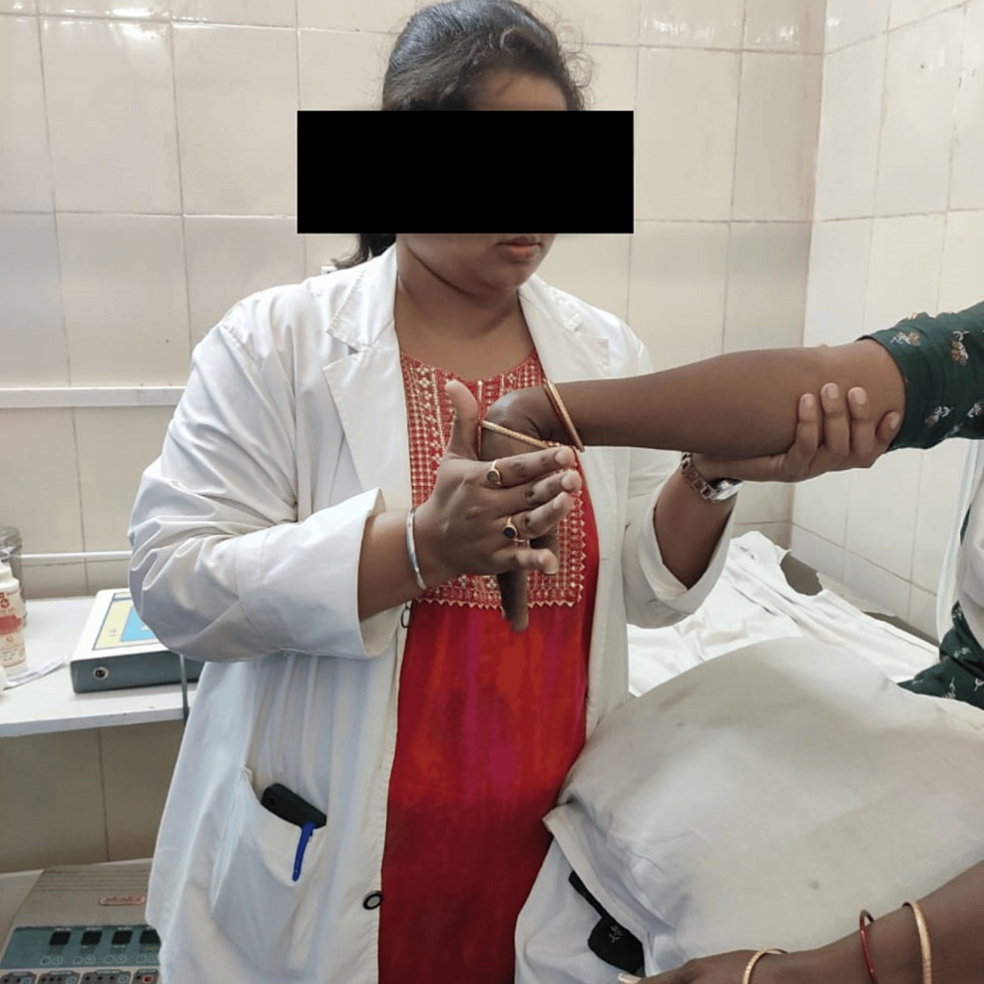
Therapist giving stretching to wrist extensor muscle

**Figure 3:**
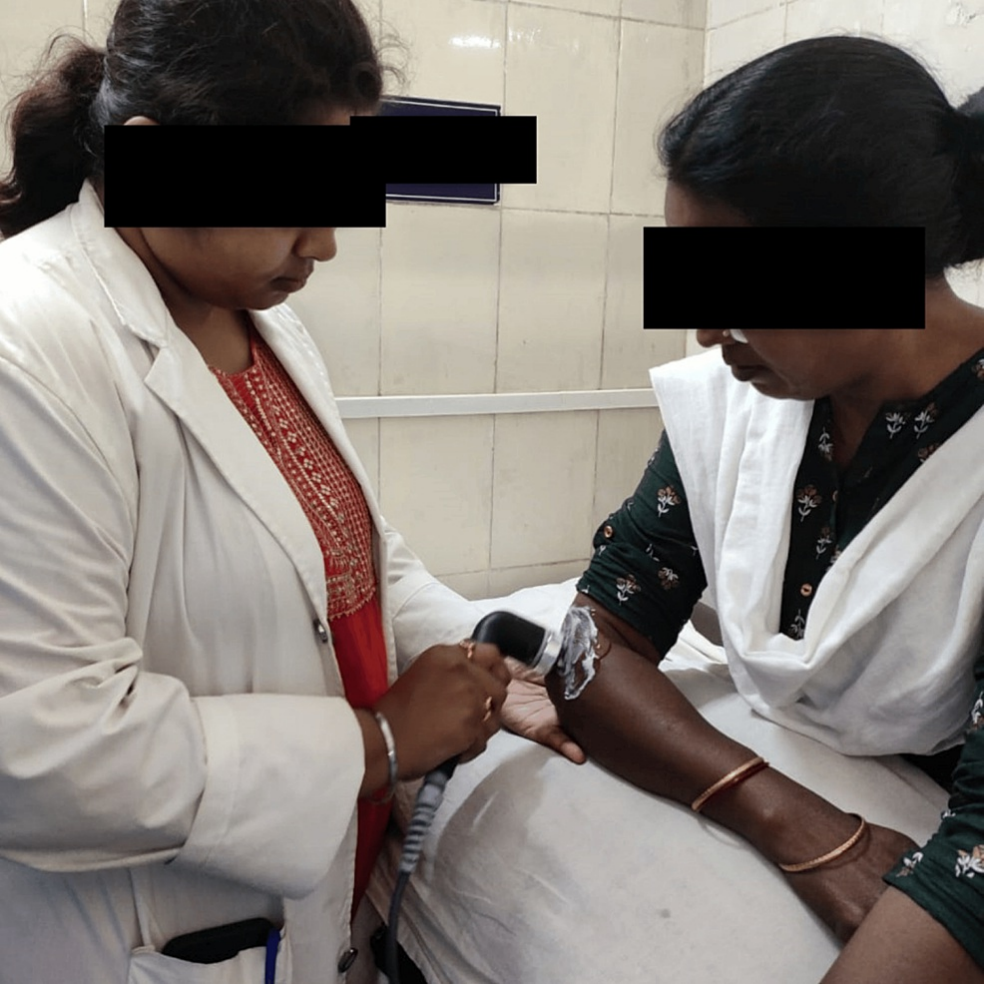
Ultrasonic therapy

Physiotherapy intervention was initiated and consisted of a week treatment plan, including the use of a soft tissue technique. The active release technique works to relieve adhesions and fibrosis that can accumulate in tissues due to overload from repetitive use, thereby reducing tissue tension with conventional management (Table 4 and Figures 1-3).

**Table 4:** Physiotherapy intervention

Intervention	Dosage
Conventional treatment, ultrasound - continuous mode, 3 MHz with an intensity of 1 W/m^2, in slow circular movements or a longitudinal pattern	3 times a week, every other day, for a total of 12 sessions spread over 4 weeks for 8 minutes
Isometric stretches for wrist joint	3 to 5-second holds and 10 repetitions/session once daily
Praying position stretches	
Stretching exercises	10 to 30-second holds and 10 repetitions × session once daily
Ice-fomentation	30 seconds × 10 stretches every session × 1 per day [15,16]
Active release technique	3 times a week for 4 weeks; a total of 12 sessions lasting 10 minutes each will be conducted [17]

Follow-up and outcome

Following four weeks of therapy, the individual underwent evaluation and showed improvement in her condition and follow-up was continued for a month. Table 5 shows outcome measures.

**Table 5:** Outcome measures VAS: Visual Analog Scale, PRTEE: Patient-Rated Tennis Elbow Evaluation.

Outcome measure	Pre-treatment	Post-treatment
VAS	7/10	2/10
PRTEE	50/100	25/100

## Discussion

As physiotherapists are known for their healing hands, this case can be a fantastic example of that, as the patient received only active release technique (ART) in addition to traditional treatment. As a result, we determined that the active release approach with conventional treatment can be utilized successfully to treat elbow problems such as lateral epicondylitis. The tendons' pathological abnormalities have been described as fibro-angiomatous hyperplasia, a word that describes low-quality, slow-healing, and painful tissue. If the patient resumes the precipitating activity before the inflammatory response has completely subsided and the patient has developed sufficient muscle strength and endurance, a recurrence is likely to occur. In chronic situations, adhesions between the tendon and the joint capsule may form [18].

The current study was to examine the impact of conventional therapy and ART on the grip strength, functional performance, and discomfort of individuals with lateral epicondylitis. Patients use a 10-point VAS, which goes from 0 to 10, to rate their level of discomfort during the last 24 hours. Its validity as a contemporaneous and predictive measure of pain intensity has been shown. The Patient-Rated Tennis Elbow Evaluation (PRTEE) scale assessed the subject's function and discomfort during the previous week [19].

A study done by Agarwal et al. in 2022 investigated whether patients with lateral epicondylitis would benefit significantly from move kinetic taping (MKT) in addition to standard conventional physiotherapy treatment in terms of grip strength and function. MKT was circumferentially placed around the wrist extensor muscle bellies, so providing general proprioceptive feedback resulted in a considerable increase in grip strength. When compared to traditional exercises, MKT resulted in a significant improvement in the function of the elbow and its grip strength, suggesting that it may one day be used in conjunction with conservative physiotherapeutic intervention to treat lateral epicondylitis. This study concludes that using MKT significantly improved the function of the elbow and its grip strength in comparison to traditional therapy. This proves that subsequent treatment for lateral epicondylitis may involve the use of MKT in addition to conventional physiotherapy [20].

## Conclusions

Tennis elbow, which causes pain and decreased grip strength, has an impact on a person's everyday activities in general. A physiotherapist is crucial in treating these overuse injuries and returning the patient to their daily activities. Following four weeks of consistent treatment, a significant enhancement was observed in the subject's elbow function and grip strength. As a result, overall function is enhanced.
